# The Effect of Magnification and Contrast on Reading Performance in Different Types of Simulated Low Vision

**DOI:** 10.16910/jemr.10.2.5

**Published:** 2016-02-28

**Authors:** Michael Christen, Mathias Abegg

**Affiliations:** Department of Ophthalmology, Inselspital, Bern University Hospital, and University of Bern, Switzerland

**Keywords:** eye tracking, low vision, reading, nystagmus

## Abstract

Low vision therapy, such as magnifiers or contrast enhancement, is widely used. Scientific evidence proving its efficacy is scarce however. The objective of this study was to investigate whether the benefits of magnification and contrast enhancement depended on the origin of low vision. For this purpose we measured reading speed with artificially induced low vision in 12 healthy subjects in conditions of a simulated central scotoma, blurred vision and oscillopsia. Texts were either blurred, set in motion or blanked at the gaze position by using eye tracking and gaze contingent display. The simulated visual impairment was calibrated such that all types of low vision caused equal reading impairment. We then tested the effect of magnification and contrast enhancement among the different types of low vision. We found that reading speed improved with increasing magnification and with higher contrast in all conditions. The effect of magnification was significantly different in the three low vision conditions: The gain from magnification was highest in simulated blur and least in central scotoma. Magnification eventually led to near normal reading speed in all conditions. High contrast was less effective than high magnification and the effect of contrast enhancement was similar in all low vision conditions. From these results we conclude that the type of low vision determines the benefit that can be expected from magnification. Contrast enhancement leads to similar improved reading speed in all low vision types. We provide evidence that supports the use of low vision aids.

## Introduction

Low vision is defined as an uncorrectable loss of vision that restricts affected patients in their everyday life. The International Classification of Diseases assumes moderate to severe vision impairment with visual acuity below 6/18 but better than 3/60 in the better eye with the best possible correction ( 32 ). According to estimations of 2010 globally 191 million people suffer from low vision ( 28 ). Major causes for vision loss are cataract, age related macular degeneration, diabetic retinopathy, glaucoma or refractive error ( 2 ).

Reading impairment is a main complaint of patients suffering from low vision. Hence improving reading speed is the primary therapeutic goal ( 6 ). Low vision therapy has a long-standing tradition and is widely pro-vided. Its aim is it to enable and support low vision pa-tients in reading and other daily activities. In order to decide which low vision aids are beneficial for an individual patient, near and distance visual acuity, contrast sensitivity and visual field are taken into account. Usually however it is difficult to predict whether and how much an individual patient may benefit from low vision therapy ( 15, 17, 31 ). This may be due to the scarce evidence from studies about the efficacy of low vision treatment, which has been examined, with some exceptions ( 26, 27 ), in small observational studies ( 29 ). Improvement of reading speed ( 20, 21 ) and quality of life ( 11, 14 ) could be found. In a randomized trial Stelmack et al. showed that training and instruction enabled significantly better performance than provision of low vision aids alone ( 22, 27 ). The great majority of studies focused on patients with macular disease causing a central scotoma. However low vision may have a great variety of causes, which raises the question whether all kinds of low vision respond equally to low vision support, irrespective of the cause.

To investigate this we compared the effect of magnification and contrast enhancement on reading performance in three different types of low vision. Measurements were performed in healthy subjects with a computer based simulation of low vision diseases. This approach gave us the possibility to compare directly and within subjects the different low vision types and furthermore we could avoid bias induced by comorbidities that may be found in real low vision patients. Low vision types tested in this study were simulated blurred vision, simulated oscillopsia and simulated central scotoma.

## Methods

### Subjects

Twelve healthy subjects with a mean age of 27 years (range 25-31 years) took part in the experiment. All were native German speakers. All subjects had a visual acuity equal or better than 20/20, tested at 0.5m. The study was conducted with approval of the local ethic committee Bern, Switzerland and all the subjects gave informed consent in accordance with the Code of Ethics of the World Medical Association (Declaration of Helsinki).

### Experimental Setup

Reading texts were presented on a 21” CRT-monitor (ViewSonic G220fb) with a refresh rate of 100Hz and a resolution of 1024 to 768 pixels. Participants viewed stimuli binocularly from a distance of 0.5m. One screen pixel corresponded thus to a visual angle of 0.045° (=146 seconds of arc). Eye movements were recorded using an EyeLink 1000 eye-tracking system (SR Research, Mississauga, Canada) with a sampling rate of 2000Hz. To stabilize the head position, a chin and forehead rest was used. On the chin rest a microphone was attached to record the participants’ voice.

**Figure 1:**
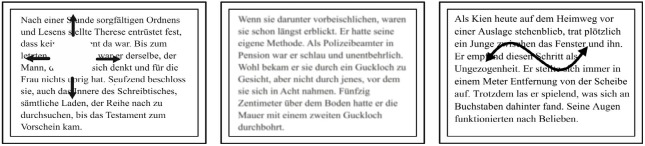
Illustration of the low vision types. Left panel, shows simulated central scotoma, middle panel shows simulated blurred vision and right panel illustrates simulated oscillopsia. To ensure all types of simulated low vision lead to the same reduced visual acuity in all subjects, a calibration was performed with adjustments of the scotoma size, the blur radius and the frequency of the oscillopsia.

To simulate a central scotoma, a white disc covering the reading text at the center of gaze was displayed (illustrated in figure 1). For this, we used the technique of gaze contingent display, which is a function of the EyeLink1000 system ( 24, 25 ). To simulate blurred vision, texts were blurred with a mean filter from the software ImageJ 1.46 (Public domain, National Institutes of Health, USA; figure 1). The mean filter replaces each pixel value in an image with the mean value of all pixels within a given radius. To simulate oscillopsia, reading texts were set in motion with a sinusoidal movement (illustrated in figure 1). The movement pattern consisted of two overlaid sine functions, one with an amplitude of 70 pixels horizontally and the other with an amplitude of 40 pixels vertically resulting in excursion of 140 pixels (6.4°) horizontally and 80 pixels (3.6°) vertically.

### Experimental Protocol

The three different simulated low vision conditions were tested in separate experimental blocks. Between these blocks, the participants took a short break. Each block started with a calibration of the eye tracker using the default nine-point calibration procedure. The calibration was validated and only accepted if the eye tracking was not off by more than a mean of 0.5° and a maximum of 1°.

In every block the parameters of the low vision simulation were adjusted such that the visual acuity was equally reduced for all participants in all simulations. To achieve this we measured visual acuity with numbers of 14 pixels size, which corresponds to a Snellen visual acuity of about 20/100 in our conditions. We then worsened the low vision conditions stepwise until the participants could not correctly identify at least 8 of 12 numbers within 30 seconds. We then used the parameters of the preceding step for the experiment. The central scotoma was adjusted by making the diameter of the disc bigger. A scotoma (disc) with a diameter of 0.5° was chosen as starting point and was then increased in step sizes of 0.5°. To calibrate the blurred vision, the test started with a blurring radius of one pixel (146” seconds of arc). This radius was then increased in steps of one pixel. In the simulated oscillopsia, we increased the frequency of the sinusoidal movement: The initial-frequency was 0.4 Hz horizontal and 1.1 Hz vertical. For each step, the horizontal movement was increased by 0.2 Hz and the vertical movement was increased by 0.3 Hz. After the calibration of the eyetracker and the adjustment of the low vision simulation were successfully completed the participants had to read texts in 6 different magnifications and 5 different contrast levels. The german texts were taken out of the novel ‘Die Blendung’( 4 ). Each text passage had a mean length of 55 words (range 48 to 62) and a mean length of 291 characters (range 272 to 309). As font we used Times New Roman. All the texts were distributed over either nine or ten lines, no matter which magnification was used.

In a series of pilot experiments we determined the range of magnification such that we were not at a floor or ceil-ing effect (data not shown). For contrast we used the range possible with our CRT screen. To test the effect of magnification a scale with a step size of 0.1 log unit (base10) was used, resulting in font sizes of 14, 18, 22, 28, 35 and 44 pixels (table 1). The magnification factor between each level was ¹⁰√10 = 1.2589 analogue to stand-ard visual acuity charts (ISO, 2009). Font sizes were rounded to whole numbers. For the contrast levels the text was presented in different shades of grey (reduced luminance) while the background was kept white (maximal luminance). Five contrast levels of 4.9, 11.9, 27.4, 56.9 and 100% corresponding to contrast sensitivity of 0, 0.4, 0.8, 1.2 and 1.6 were chosen for the experiment (table). Contrast C was calculated by using following formula: C=10^-S^. The Michelson-equation C = (L_max_- L_min_)⁄(L_max_ + L_min_) was used to determine the ratio between the minimal and maximal luminance given the contrast C: (1-C)⁄(1+C)= L_min_⁄L_max_. The texts were saved as 8-bit greyscale images: In those 0 is defined as black and 255 is defined as white. Given L_max_=255 (white background), the text luminance L_min_ was calculated by 255∙(1-C)⁄(1+C)=L_min_. All texts of the contrast levels were presented at a font size of 18 pixels (0.81°), which corresponds to the second magnification level. The participants were instructed to read the text aloud and as fast and as correct as possible. Words should be repeated to correct mistakes. As soon as the last word was spoken, the time was manually stopped by the examiner by pressing a key. The maximum allowed time for one trial was 60 seconds, it was aborted after that. In order to counter-balance learning and fatigue effects the order of the three simulations conditions were varied and the levels within one simulation were randomized. To prevent effects from variations in difficulties of the texts, they were allocated different to the testing levels for every participant. Addi-tionally to the main experiment, we tested the reading speed without any visual impairment for the same texts with the same magnification and contrast levels in four of the twelve subjects. This test with normal vision was performed separately from the remaining experiment.

**Table 1:** Size and contrast characteristics of the tested levels. The value of the text luminance is derived from an 8-bit image where 0 is defined as black and 255 is defined as white.

Magnification level	Font size [pixels]	Font size [degree]	ContrastC [%]	Text luminance L
1	14	0.63	27.4	185
2	18	0.81	27.4	185
3	22	0.98	27.4	185
4	28	1.25	27.4	185
5	35	1.57	27.4	185
6	44	1.97	27.4	185
Contrast level				
1	18	0.81	4.9	243
2	18	0.81	11.9	225
3	18	0.81	27.4	185
4	18	0.81	56.9	110
5	18	0.81	100	0

### Analysis

For each trial, incorrect words (i.e. words that were not correctly read or not read within 60 seconds) were counted manually after the experiment by listening to the recordings. The number of characters of these words were subtracted from the total number of characters of the text in order to get the number of correctly read characters in a given trial. The reading speed was then expressed in correctly read characters per second *c*⁄*s* : read characters per second [c⁄s] = (total given characters [c]-incorrect read characters[c])⁄ (reading time [s]). For statistical analysis we used a linear mixed effects model with reading speed as dependent variable. Low vision condition (central scotoma, oscillopsia, blur) and level of magnification (14, 18, 22, 28, 35, 44) or contrast (243, 226, 186, 110, 0) were used as independent variables. Subjects were used as random effects. To select between different fitting models (random-intercept, random-slope, or combined) we used Akaike’s Information Criterion (AIC) and chose the best model by the principle ‘smaller-is-better’. F-statistics, p-values and the Bayesian information criterion (BIC) are reported. Statistical significance was assumed if p<0.05. Analyses were performed using the MIXED procedure in SPSS (IBM SPSS Statistics 21).

## Results

**Figure 2:**
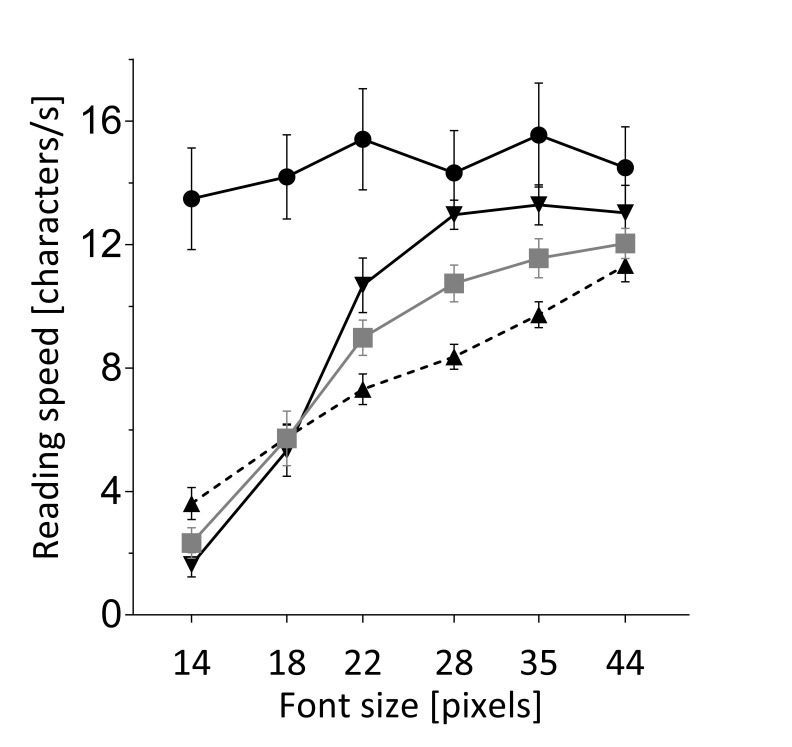
The effect of magnification on reading speed. Six magnification levels were tested with normal vision (●), simulated blurred vision (▼), simulated oscillopsia (■) and simulated central scotoma (▲). Reading speed improved with magnification in all low vision conditions, while with normal vision reading speed remained at a maximum. The effect of magnification depended on the low vision type. Error bars indicate standard error of the mean.

**Figure 3:**
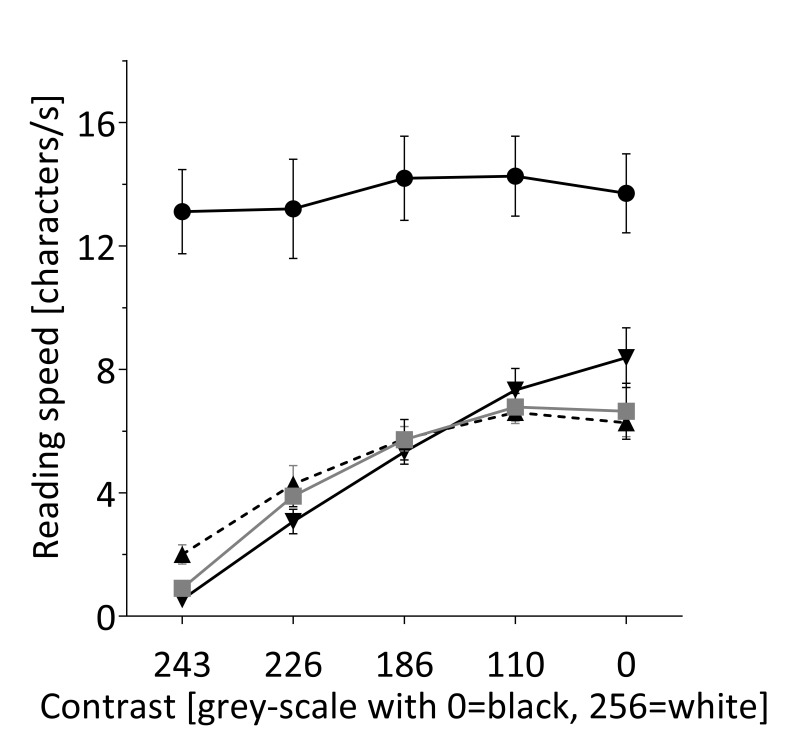
The effect of contrast enhancement on reading speed. Five contrast levels were tested with normal vision (●), simulated blurred vision (▼), simulated oscillopsia (■) and simulated central scotoma (▲). As for magnification, the reading speed improved with enhanced contrast in all low vision conditions but not for normal vision. The effect of enhanced contrast was not statistically significant between the different low vision conditions. The maximal reading speed with the highest contrast remained below the normal reading speed. Error bars indicate standard error of the mean.

The calibration of the low vision condition was chosen such that a letter requiring a Snellen visual acuity of about 20/100 was just visible (see methods for details). To achieve this with a central scotoma, a blanked area with a diameter of 3.16° (range 2.5° – 4.0°) was necessary. For the same vision a blur radius of 329 seconds of arc (range 292 – 438 seconds of arc) was necessary. To induce the same reduction of vision a horizontal movement speed of 1.3Hz (range 1.0 – 1,6Hz) and a vertical movement speed of 2.45Hz (range 2.0 – 2.9Hz) was required. Next we measured reading speed and we found that in all types of simulated low vision reading speed improved with magnification (oscillopsia: F(5,55)=82, p<0.001, BIC=284; blur: F(5,55)=90, p<0.001, BIC=308; central scotoma: F(5,50)=55, p<0.001, BIC=236; figure 2) and in all low vision conditions reading speed improved with better contrast (oscillopsia: F(4,44)=30, p<0.001, BIC=249; blur: F(4,44)=65,p<0.001, BIC=238; central scotoma: F(4,40)=16, p<0.001, BIC=213, figure 3). In contrast to subjects experiencing low vision, the reading speed in subjects with normal vision did neither change with contrast (F(4,12)=0.9, p=0.5, BIC=69) nor with magnification (F(5,15) = 2.4, p = 0.1, BIC=77). At the second but lowest magnification and contrast condition reading speed was similar in all three conditions (magnification: F(2,33)=0.1, p=0.9, BIC=167; contrast: F(2,33)=1.0, p=0.4, BIC=154), indicating that all types of low vision led to a comparable impairment of reading at this particular contrast and magnification. We found that the reading speed with the highest magnification was similar to the reading speed measured in normal viewing conditions (F(1,37)=3, p=0.1, BIC=177) and it was significantly better than the reading speed with the highest contrast (F(1,68)=72, p<0.001, BIC=328). The reading speed with the latter was significantly below the reading speed measured in normal viewing conditions (F(3,35)=9.8, p<0.001, BIC=176).

Importantly we found a significant interaction term of magnification and low vision condition (F(2,193)=8.2, p<0.001,BIC=947), showing that the effect of magnifica-tion on reading is significantly different among the three conditions: Little increase in magnitude led to the most improvement of reading speed if low vision was induced by blur, where the benefit plateaued at a font size of 28 pixels. In contrast, low vision induced by a central sco-toma showed a more gradual increase of reading speed with increased magnitude, while the effect of magnifica-tionon low vision induced by text movement was at an intermediate level. Unlike for magnification we found that the interaction term of contrast and low vision condition showed no significance, indicating that the benefit of reading speed from enhanced contrast is not significantly different between the three low vision conditions (F(2,158)=0.9, p=0.39, BIC=897).

## Discussion

We found that artificially induced low vision led to reduced reading speed that could be improved with both magnification and better contrast. We found a near normal reading speed with the highest magnification but not with the best contrast in all types of simulated low vision. Importantly we found that the effect of magnification significantly depended on the type of low vision: The best effect of magnification was found for blur associated low vision, followed by low vision associated with text motion. The least effect was found for subjects with a simulated central scotoma. Contrast enhancement on the other hand, showed benefits that were independent from the type of vision loss.

Even though the reading speed with small text size was not different in the three conditions, the same increase in magnification led a bigger effect for blur associated low vision than for central scotoma and nystagmus. This observation is to our knowledge unprecedented and the reasons for this are unknown. We speculate that the rate limiting factors for reading may be different in the three conditions and these factors may respond differently to magnification. For example, two-point discrimination may be the rate limiting step in the blur condition, which prevents to differentiate the letter “U” from the letter “O” for example. A small increase in size will enable this differentiation and thus facilitate reading. In contrast to blur, the number of obscured letters may be critical for reading with a central scotoma. Possibly the magnification required two recognize more letters of a word is more than the magnification required to improve two-point discrimination. Similar speculations could be made for oscillopsia and reading with nystagmus.

Where data is available, our results correspond well with findings from patients, where too a benefit on reading speed was found with magnification( 18 ). While we found a near normal reading speed with the highest magnification for all low vision conditions, Legge et al. found near normal reading speed only in patients with intact central vision while patients with loss of central vision only reached a median maximal reading speed of 25 words/minute( 18 ). Letters with font size between 12° and 24° were used in the latter condition, while the font size was about 2° in our study. Thus our results may only be applied to patients without loss of central vision as an optimal font size beyond 2° is usually associated with reduced maximal reading speed( 16 ). The finding of near normal reading speed with the highest simulated magnification in all simulated low vision types confirms our previous finding of near normal reading speed in both patients with nystagmus and simulated nystagmus( 5 ). In another study by Barot et. al. about reading speed in 71 patients with infantile nystagmus normal reading speed was found for most patients if texts were provided with optimal font size. Only the group of patients (n=12) with visual acuity below or equal 20/80 showed slightly reduced reading speed( 1 ). Again, all these findings indicate that up on a certain amount of visual impairment reading speed is not limited if text is presented in optimal conditions( 18 ).

Also with regard to contrast our data agree with data obtained from patients. We too found an improved reading speed with increased contrast as others had found in patients( 9, 23 ). Data from patients showed that the effect of contrast enhancement depended on individual contrast sensitivity and that it is independent of the type of low vision( 23 ). These clinical results correspond well to our finding of an absent effect of the type of low vision on contrast enhancement. Giacomelli et al. found that contrast reduction at a given text size led to more pronounced impairment of reading in patients with advanced low vision than in patients with mild low vision( 9 ), thus suggesting that contrast depends on the amount of visual loss. We found that contrast affected reading speed barely if no low vision was present. In a low vision situation, i.e. about 20/100, reduced contrast affected reading significantly however. Both findings indicate that patients with low vision have a smaller bandwidth of contrast where optimal reading is possible than subjects with normal vision.

Simulating visual impairment, especially simulating central field loss with the technique of gaze contingent display has been used by many researchers in the past. Research has been conducted on visual search with central scotoma( 8, 30 ), visual sensitivity in peripheral vision( 13 ) or on reading with central scotoma( 3, 10 ). There is virtually no literature on the effect of magnification and contrast on reading in simulated low vision however. In one study of Fine & Rubin reading speed was measured with different magnifications in three subjects with simulated central scotoma, simulated cataract and both combined( 7 ). They found that if the two low vision conditions were combined, more magnification was needed to reach the same reading speed as when only one low vision condition was simulated. Similar to our results, they also found that simulated cataract had almost no impact on reading performance with large letters.

The simulated low vision as used in the current paper has limitations which make a direct comparison with affected patients difficult. The simulated conditions differ from affected patients in several respects. First, in our conditions subjects are tested in an unadapted state, ie. subjects had no time to adjust to the situation. It is possible that reading speed in conditions of a central scotoma would improve over time, i.e. once subjects develop a new preferred retinal locus or learn to optimally use a pseudofovea (for an overview see for example ( 19 )). Second, we tested in a group of young subjects whereas affected patients are usually older. Third, low vision from ophthalmic disease itself is not directly comparable to the simulated low vision. The shape of the central scotoma is not a circle in real life, a nystagmus has a motor component, which probably interacts with the sensory component of oscillopsia and blur in a patient may not be gaussian but may rather result from higher order optic aberrations. All this prevents a direct and quantitative comparison of our findings with data from patients. And yet we are convinced that the use of a simplified model allows correct qualitative data which also apply to patients and thus our main conclusion that different causes for low vision respond differently to low vision aids is valid. To prove this in a group of patients will be difficult though because different types of low vision are associated with different types of age, genetic background and comorbidities thus making an investigation of the low vision aid effect alone difficult.

Taken together, our data strongly support the use of magnification and contrast enhancement in all types of low vision. Our results suggest that for a given visual acuity and a given visual impairment the benefit of low vision therapy depends on the cause. This advocates measures that are adjusted to the origin of low vision, which today is achieved empirically( 15, 18 ).

## Acknowledgement and declaration of conflict

This study was supported by the Swiss National Science Foundation. The authors report no conflicts of interest and have no proprietary interest in any of the materials mentioned in this article.
